# Polish Emergency Dispatchers During a COVID-19 Pandemic – Burnout Syndrome, Perceived Stress, and Self-Efficacy. Effects of Multidimensional Path Analysis

**DOI:** 10.3389/fpsyg.2021.729772

**Published:** 2021-10-08

**Authors:** Marta Makara-Studzińska, Maciej Załuski, Katarzyna Adamczyk

**Affiliations:** Department Health Science, Institute of Nursing and Midwifery, Jagiellonian University Medical College, Kraków, Poland

**Keywords:** emergency service, self-efficacy, occupational burnout, perceived stress, COVID-19, emergency call-taker and dispatcher

## Abstract

International research has demonstrated that emergency call operators face unique risks to their mental health, in particular job stress, and occupational burnout syndrome. There is already wide knowledge about the relationship between stress, burnout and employee personal resources, which has practical application in preventing mental health. However, more research into the subtle relationships between variables is needed. The aim of the study was to check the moderation effect of differences in the intensity of latent variables on the relationship between perceived stress, self-efficacy and professional burnout. The participants were 546 call-takers and dispatchers from 14 public-safety answering point in Poland aged between 19 and 65 years. The Link Burnout Questionnaire, the 10-item Perceived Stress Scale, the Generalized Self-Efficacy Scale, and an independent questionnaire were used to gather information. The method of path analysis was used. The study confirmed the existence of negative relationships between perceived stress (assessment of the current situation) and self-efficacy (a personal trait). Taking into account the moderating effect of latent variable: psychological comfort revealed a hidden relationship between stress and burnout. The stress-burnout relationship occurred only among participants with low level of psychological comfort, so it was not a proportional relationship. In the case of participants with a high level of second latent variable: power-to-affect, the hypothesis that a high level of this variable should weaken the relationship between stress and burnout was not confirmed. The level of latent variables did not affect the self-efficacy relationship with occupational burnout. Taking into account the differences in the intensity of latent variables showed their moderating effect, which often turned out to be different from the assumed one and obtained in the research of other authors. This allowed to discover the relationships that might otherwise have been overlooked and not included in burnout prevention. The results showed a high level of occupational burnout in the ECD’s group during the COVID-19 pandemic: 32% of the responders reported emotional exhaustion, 53% loss of professional effectiveness.

## Introduction

Emergency call-takers and dispatchers (ECDs) are a key component of emergency care. Both in Poland and in other countries, this profession is highly fluid. One of the reasons is that work exposes the employee to a number of strong stressors. These include high responsibility for the safety of reporting individuals and for the health of the emergency personnel. American research has shown that 42% of operators assess their work as “stressful and very stressful,” 47% “demanding,” and 14% “extreme demanding,” regardless of gender and length of service (Meischke et al., 2015). ECDs must quickly identify specific constellations of risk indicators for human health and life through the collection of critical information and the effective delivery of appropriate first aid instructions. The health and life of another person may depend on the correct selection and evaluation of the obtained information (Forslund et al., 2004; Meischke et al., 2010). Although the contact of the emergency number operator with traumatic stressors is made by phone, it is as stressful as direct contact (Golding et al., 2017; Kindermann et al., 2020). Conversation with a traumatized person may give the operator symptoms of peritraumatic stress, secondary post-traumatic stress (secondary traumatic stress, STS), PTSD and other anxiety disorders as well as depressive symptoms (Baseman et al., 2018; Klasa, 2020). It is also noted that some personal characteristics of operators may contribute to negative health changes. Excessive commitment to work, ways of coping with work stress, exhausting psychophysical forces have been indicated as predictors of stress at work ECD (Meischke et al., 2015).

According to the transactional theory of stress, every stress reaction begins with an assessment of the current situation (Folkman, 2011). Perceived stress is an outcome variable-measuring the experienced level of stress as a function of objective stressful events, coping processes, personality factors (Cohen et al., 1983, p. 386). Individuals with high level of perceived stress considered their lives as unpredictable, uncontrollable and overloading (op, cit, p. 387). Work-related stress belongs to the broad group of psychological risks, which have the potential to cause psychological and physical issues (Chirico, 2015). Cox et al. (2000) distinguished 10 categories in order to characterize sources of work-stress in the social and organizational context of work. Among the potential sources of health risks are mentioned: combined exposure to physical and psychosocial risk, job insecurity, high emotional load related to burnout and others (Chirico, 2017b). Psychosocial risks follow changes in the work environment, the economic situation of countries, changes in the labor market and the effects of random factors (SARS-CoV-2 pandemic). This fact forces the continuous improvement and evolution of the definition of psychosocial risks. Following the definition of health adopted in 1986 by the World Health Organization (WHO) as “positive state of complete physical, mental and social well-being” (Nutbeam, 1986), in which work-related stress is treated as a significant health risk factor. The social and economic costs of work-related stress from employee absenteeism, health and social care and loss of productivity are high worldwide (Chirico, 2017b). Predictors of stress at work of ECD are also: young age, female gender, lower level of education and lack of social and family support (Kindermann et al., 2020). Environmental and individual factors can buffer the negative effects of stress. It is believed that the following personal resources: self-efficacy, resilience, and empathy, may contribute to the reduction of stress related to the work of ECD’s (Baseman et al., 2018). Self-efficacy refers to an “beliefs in one’s capabilities to organize and execute the courses of action required to produce given attainments” (Bandura, 1997, p. 3). High levels of self-efficacy are associated with goal setting, persistence, and a constructive way of dealing with failures (Schwarzer and Renner, 2000). Furthermore, self-efficacy enables individuals to trust their capabilities and to face stressful demands with confidence (Jerusalem and Mittag, 1995).

Occupational burnout is a psychological syndrome emerging as a prolonged response to chronic interpersonal stressors on the job (Maslach and Leitner, 2016, p. 103). Recently, there has been a change in the way WHO defines the occupational burnout syndrome. Until now, burnout was understood as “state of vital exhaustion” belonging to the group “problems related to life management difficulty” (World Health Organization [WHO], 2011; Chirico, 2017a). According to the definition that will come into force in January 2022, burnout is a syndrome conceptualized as resulting from chronic workplace stress that has not been successfully managed. It is characterized by three dimensions: feelings of energy depletion or exhaustion; increased mental distance from one’s job, or feelings of negativism or cynicism related to one’s job; and reduced professional efficacy (World Health Organization [WHO], 2019). The new definition encourages the differentiation of burnout from symptoms of other mental disorders: depression, anxiety and stress (Chirico, 2017a). The distinction in the definition of three dimensions of burnout draws attention to a number of psychological risks occurring in the work environment, which cannot be identified as the stressors of the work environment alone (Chirico, 2015). After the change, occupational burnout will not be classified as a disease state, but as an “occupational phenomenon” – a strictly professional problem related to the experiences of an employee emerging in the context of professional work. Meanwhile, according to many researchers, there is scientific evidence for treating the burnout syndrome as occupational disease (Chirico, 2017a; Laštovková et al., 2018). Burnout refers specifically to phenomena in the occupational context and should not be applied to describe experiences in other areas of life. Occupational burnout syndrome has its own specific antecedents and consequences, different from the work-related stress risk factor, which can only be explained by new theoretical models (Chirico, 2017b). The Job Demands—Resources (JD-R) Model of Burnout (Demerouti et al., 2001) is one of models explaining the mechanisms of occupational burnout and work commitment. Professional work influences energy levels, which are responsible for enhancing experienced stress and lead to a depletion of psychophysical resources and the deterioration of health. Energy processes are modified by the motivational processes related to the possessed resources (e.g., self-efficacy), which protect against the development of burnout. Occupational burnout syndrome is a result of the depletion of mental and psychical energy as well as the cognitive resources of a person (Grabowski et al., 2019). The symptoms of occupational burnout develop as a consequence of an overload caused by workplace requirements until a person’s psychophysical resources are depleted, which in effect decreases their motivation to engage with their work. Other model of burnout based on the conservation of resources (COR) theory assuming that burnout is an effect of stress, which occurs when individuals perceive that the resources, they value are threatened. Following this theory, both perceived stress and self-efficacy can be treated as human resources that are interrelated in the so-called “caravans” of resources (Hobfoll et al., 2018). Stress is an element of the human resource protection system, and also a signal for the perceived threat of losing resources. However, a prolonged presence of stress can lead to a spiral of further losses. In the course of professional work, losses can manifest themselves in the form of signs of burnout that appear successively, ranging from emotional exhaustion to disappointment with the job (Jaworowska, 2014; Rogala et al., 2016). Symptoms of the burnout syndrome are indicated as the main cause of low retention in operator positions and high sickness absence (Baseman et al., 2018). Operators report lower job and life satisfaction compared to other employees (op, cit.).

Job Demands—Resources Model and COR theory treat self-efficacy as one of the important personal human factors, which protect against the development of burnout (Hobfoll, 2011; Grabowski et al., 2019). Scientific research shows that the role of self-efficacy increases when work requirements threaten to lose human energy resources, e.g., in a situation accompanied by an increased level of stress (Rogala et al., 2016). Relationships linking stress, self-efficacy, and burnout have been studied for many years. The literature underlines to the importance of self-efficacy in promoting mental health in emergency services employees (Shakespeare-Finch et al., 2015). The research results show that stress usually remains positive to the level of occupational burnout, and negative to the level of self-efficacy (Kelley and Gill, 1993; Demerouti et al., 2001). The relationships: self-efficacy – occupational burnout are most often also negative (Grau et al., 2012; Shoji et al., 2015). Self-efficacy acts as a buffer to protect the human body against burnout. The direct effect of beliefs about self-efficacy on human well-being and job-functioning is fairly well documented, however, there is less research on their indirect functions. Researchers analyzed both the mediating and moderating functions of beliefs. The mediating function is better documented (Baka and Cieślak, 2010). Jex and Gudanowski (1992) found no empirical evidence of indirect effects of self-efficacy, in the processes of stress and burnout. However, some authors (Jex and Bliese, 1999) consider that self-efficacy is relevant to stress by serving a moderating role. We propose that the so-far unknown mechanisms or variables may hide the differences in the results. Similarly, the role of perceived stress is not resolved, which can be treated both as a human resource and a manifestation of the adaptation process, as well as an expression of failure in the process of coping with adversities.

Can differences in the levels of perceived stress and self-efficacy moderate the relationships between the variables? Our study examined the impact of differences in the severity of explanatory variables on the relationship between stress, self-efficacy and burnout. We adopted the assumption to treat the perceived stress as a categorical variable which, depending on its characteristics (high – medium – low), differentiates the relationship of stress with self-efficacy and burnout. Similarly, we found that there was a rationale for considering the differences between high, medium, and low self-efficacy levels. Differences in levels affect the form of the self-efficacy relationship with stress and burnout. We were followed the information from the scientific literature showing differences in the levels of stress in patients with different somatic ailments (Juczyński and Ogińska-Bulik, 2009). Similarly, there are differences in the intensity of the self-efficacy personality trait among patients with different health problems, behavioral disorders, or mental disorders. We assumed that the results of the measurement of perceived stress can be grouped in the form of a latent variable which we called psychological comfort. The psychological comfort, measured as low level of perceiving stress, may be interpreted as a state of calmness, absence of anxiety and tension, attentiveness, which is an effect of one’s perception regarding the content of cognitive beliefs about oneself during the process of appraising stressful situations. Low effectiveness in coping with existing problems leads to symptoms of low psychological comfort (measured as high level of perceived stress). Knowing about the level of psychological comfort helps to plan actions to protect against the negative impact, especially that of chronic stress on cognitive functions and against the development of clinical symptoms. Differentiation in level of self-efficacy (high – moderate – low) has been operationalized as a latent variable under the name: level of power-to-affect. Employees with a high level of self-efficacy have confidence that they can use their skills effectively to manage with job tasks, job challenges, and job-related stress (Shoji et al., 2015). Workers with low levels of self-efficacy have beliefs that they cannot protect themselves from negative outcomes of job stress and job-challenges. We created two models that were used to test the following hypotheses:

Model 1.

1. The level of psychological comfort moderates the negative relationship between stress and self-efficacy. If the comfort level is low, the relationship is stronger.
2. The level of psychological comfort is moderated by the positive relationship between perceived stress and burnout. A low level strengthens the relationship between the variables.
3. The level of psychological comfort moderates the negative self-efficacy relationship with burnout. If the comfort level is low, the relationship is stronger.

Model 2.

1. The power-to-affect level moderates the negative relationship between perceived stress and self-efficacy. If the power-to-affect level is high, the relationship is stronger.
2. The power-to-affect level moderates the positive relationship between stress and burnout. If the power-to-affect level is high, the relationship is weaker.
3. The power-to-affect level moderates the negative self-efficacy relationship with burnout. If the power-to-affect level is high, the relationship is stronger.

## Materials and Methods

### Participants

The data for this study were collected between January – May 2020 among all emergency call operators working in 17 public-safety answering points (PSAP) in Poland. The participants were sampled with the help of psychologists employed by each PSAP. The respondents completed an anonymous set of questionnaires which were mailed to their workplace. In total, 800 sets of research tools consisting of information about the study, 3 standardized research questionnaires, a demographic survey, and an invitation to participate were delivered. Participation in the study was voluntary. 558 sets of questionnaires from 14 PSAP in Poland were returned of which 546 were correctly completed, for a valid rate of 68,25%. The study design and protocols were analyzed and approved by the Ethics Committee of Jagiellonian University (decision No. 1072.6120.23.2017) and was carried out in accordance with the recommendations of the APA Ethics Code.

### Instruments

#### Burnout

The level of burnout was assessed using the Polish version of the Link Burnout Questionnaire (LBQ) created by the Laboratory of Psychological Tests of the Polish Psychological Society (Jaworowska, 2014). The measure consists of 24 items in relation to which the subject responds on a 6-point Likert scale [1 – never, 2 – rarely, 3 – once (or more) during a month, 4 – more or less once a week, 5 – several times a week, and 6 – every day]. The questionnaire has 4 subscales according to 4 dimensions of occupational burnout: psychophysical exhaustion (PE), relationship deterioration (RD), professional inefficacy (PI), and disappointment (DI). Each subscale measures a range between low (6 points) and high (36 points) severity. The LBQ includes 5 indicators: the higher the score of each subscale, the greater the intensity of each 4 dimensions of burnout. An additional, 5 indicators of burnout is the total burnout result, the so-called occupational burnout syndrome composite index (LBQ^*INDEX*^). It is the sum of the results obtained on the 4 subscales of the questionnaire. The Polish version of the LBQ questionnaire has good psychometric properties. The scale of DI (Cronbach’s α = 0.84) has the highest internal reliability and the scale pertaining to the PI (Cronbach’s α = 0.68) has the lowest internal reliability. In our research, Cronbach’s α for individuals ranged from PI = 0.628; RD = 0.689; PE = 0.845; and DI = 0.859.

#### Level of Perceived Stress

The Polish version of the Perceived Stress Scale (PSS-10; Juczyński and Ogińska-Bulik, 2009) was used. The PSS-10 questionnaire is the most widely used psychological instrument for measuring the perception of the cognitive aspects of stress and coping – appraising the effectiveness of coping strategies. The PSS-10 is designed to measure the level of perceived stress in terms of unpredictability, lack of control, and overload (Juczyński and Ogińska-Bulik, 2009). Ten questions of PSS-10 identify the level of perceived stress as an indicator of the effectiveness of dealing with life events. The questions in the PSS-10 ask about feelings and thoughts over the past month to which the respondent answers on a 5-point Likert-type scale: 0 – never, 1 – almost never, 2 – sometimes, 3 – quite often, and 4 – very often. The overall raw result ranges between 0 and 40 points. The PSS-10 includes one indicator: the higher the score, the greater the intensity of the perceived stress. The results also allow prediction of the physical and mental discomfort of the respondents. In the Polish version, the scale has obtained very good psychometric properties with a Cronbach’s α value of 0.86. The Cronbach’s alpha in our research was 0.883.

#### Self-Efficacy

To measure beliefs about self-efficacy in the group of emergency call operators, a Polish language version of the Generalized Self-Efficacy Scale (GSES) was used (Juczyński, 2001). The questionnaire based on Bandura’s theory of social learning measures the strength of an individual’s general belief of the effectiveness in coping with difficult life situations and smaller, daily hassles. Self-confidence in the tool tests, together with skills and knowledge, favor better coping in everyday life. The GSES consists of 10 questions to which the participant responds on a 4-point Likert-type scale: 1 – not, 2 – probably not, 3 – probably yes, and 4 – yes. The overall raw result scores were between 10 and 40 points. The GSES includes one indicator: the higher the score is, the greater intensity of the generalized self-efficacy it indicates. Cronbach’s α in the Polish version was 0.85. The Cronbach’s α in our study was 0.883.

### Procedure

The data for this study was collected between January – May 2020 among emergency call operators working in PSAP in Poland. The respondents completed an anonymous set of questionnaires which were mailed to their workplace. In total, 800 sets of research tools consisting of information about the study, three standardized research questionnaires, a demographic survey, and an invitation to participate were sent. Participation in the study was voluntary. After choosing appropriate subgroups participant completed sets of questionnaires. 558 sets of questionnaires were returned of which 546 were correctly completed. The questionnaires came from call-takers and dispatchers working in 14 out of 17 PSAP in Poland.

### Data Analyses

The authors used software environment for statistical computing R (version 4.0.5) for statistical analysis (R Core Team, 2021). A significance level of 0.05 was adopted in the analysis. Correlations between quantitative variables were analyzed using the Spearman R coefficient. The method of path analysis was used, which was performed in the R program, version 4.0.5 with the lavaan package (Rosseel, 2012).

## Results

The participants consisting of 238 male (43.6%) 308 female (56.40%). The mean age was 34.37 (*SD* = 8.14; min = 19.0, max = 65.0), 232 (42.5%) of the participants were married, 143 (26.2%) cohabited with a partner, 120 (22%) single, 44 (8%) divorced, 3 (0.6%) widowed, and 8 (0.7%) marital status was unknown. With regards to education, 396 (72.5%) of the operators had bachelor’s and master’s degrees, 143 (26.2%) had secondary education, 1 (0.18%) had vocational education, and the level of education of 6 (1.1%) participants was unknown.

### Model 1. The Level of Psychological Comfort as Moderator of Predictors of Occupational Burnout

**FIGURE 1:**
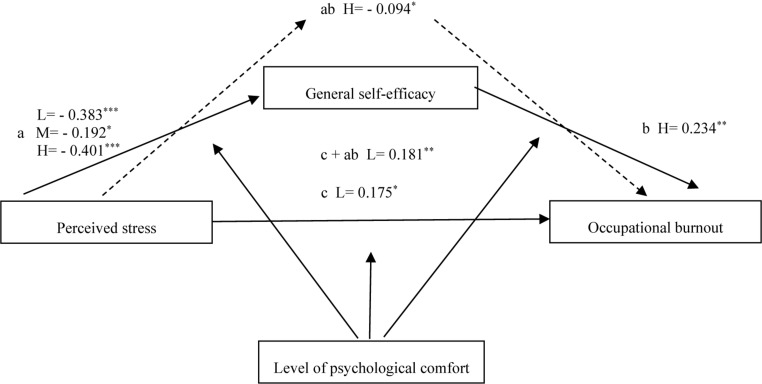
Model 1. Relationships between perceived stress, general self-efficacy and occupational burnout moderated by level of psychological comfort in a group of Polish ECD’s. L = low level of psychological comfort. M = medium level of psychological comfort. H = high level of psychological comfort. ^∗^β is significant at the 0.05 level. ^∗∗^β is significant at the 0.01 level. ^∗∗∗^β is significant at the 0.001 level. For the readability of the graph, the graphic markings of irrelevant paths were omitted.

As shown by the results presented in Table 1, the mean group value of perceived stress was 15.95 points, which corresponds to the upper limit of the moderate range. This score is 0.67 points lower than that obtained in a representative group of 1,830 randomly selected, healthy Poles (*M* = 16.62, *SD* = 7.50; Juczyński and Ogińska-Bulik, 2009), but 1.09 points higher than that obtained in the studies of 580 Polish firefighters (*M* = 14.86, *SD* = 5.72, and Cohen’s *d* = 0.17; Makara-Studzińska et al., 2019). The mean value of the self-efficacy measurement was 31.36 points and was 4.04 points higher than that obtained in a representative group of 496 randomly selected, healthy Poles (*M* = 27.32, *SD* = 5.31, and Cohen’s *d* = 0.86). The average result of the measurement of occupational burnout in the ECD’s group expressed using the composite LBQ^*INDEX*^ was 82.67 points and it was 28.31 points higher than that obtained in the group of 88 Polish air traffic controllers (*M* = 54.36, *SD* = 16.07, and Cohen’s *d* = 2.16) and 17.74 points higher than that obtained in the group of 54 sea navigators (*M* = 64.93, *SD* = 14.03, and Cohen’s *d* = 1.49; Makara-Studzińska et al., 2020). The results obtained in the measurement of each of the burnout dimensions were higher than those obtained in the standardization studies of the LBQ questionnaire in various professional groups, as well as in the studies of Polish firefighters (Jaworowska, 2014; Makara-Studzińska et al., 2019) and were within the scope of high scores in the case of three dimensions, and in the scope of loss of professional effectiveness within the scope of very high scores. A high level of burnout in the PE dimension was diagnosed in 32% of the responders, in the RD dimension in 28,6% of the responders, in PI dimension in 53,11% of the responders, and in the DE dimension in 8,97% of the responders. The sten scores adopted in the interpretation of the PSS-10 results were used to distinguish three groups of respondents due to the latent variable – psychological comfort. The group with low psychological comfort was composed of results in the range of 7–10 sten score, moderate 5–6 sten score and high 1–4 sten score. Table 2 shown the distribution of the variable “level of psychological comfort.” Nearly 32% of the examined ECD’s reported the presence of signs of high intensity of perceived stress (low level of psychological comfort) in the month preceding the study. Table 2 shown correlations among perceived stress, occupational burnout and self-efficacy. The results show that the level of perceived stress was moderately strongly negatively correlated with the level of self-efficacy and weakly positively correlated with the sense of loss of professional effectiveness. The level of self-efficacy weakly negatively correlated with the sense of loss of professional effectiveness and weakly positively with the sense of disappointment with the work performed. As each of the explanatory variables significantly correlated with at least two other variables, there was no reason to remove any of them from the model at this stage of the analysis. The path analyzing model was proposed to explain the moderating influence of psychological comfort on the relationship between explanatory variables and burnout (see Table 3 and Figure 1). As the tested models with moderation operate at degrees of freedom (df = 1), this gives rise to a very low RMSEA value and a very high CFI value. For this reason, it is not recommended to provide the values of measures of its fit (in our model RMSEA = 0, CFI = 1). Similarly, the chi-square statistic = 0 and has zero degrees of freedom.

**TABLE 1:** The correlations among perceived stress, occupational burnout and self-efficacy.

Factors	*M*	*SD*	Spearman’s coefficient						
			PSS10	GSES	PE	RD	PI	DI	LBQ^*INDEX*^
PSS10	15.95	6.76	–	–					–
GSES	31.16	4.14	−0.537***	–					–
PE	20,94	3,74	0.031	0.03	–				
RD	20,13	4,10	0.049	0.033	0.353***	–			
PI	23,04	3,06	0.217***	−0.109*	0.038	0.193***	–		
DI	18,54	3,32	0.074	0.126**	0.29***	0.239***	0.148***	–	
LBQ^*INDEX*^	82.67	9.30	0.053	0.051	0.66***	0.722***	0.468***	0.629***	–

*N = 546.*

***Statistical significance < 0.001; **statistical significance < 0,01; and *statistical significance < 0.05.

*GSES, General Self-Efficacy Scale; PSS-10, Perceived Stress Scale; LBQ, Link Burnout Questionnaire; PE, psychophysical exhaustion; RD, relationship deterioration; PI, sense of professional inefficacy; DI, disillusion; and LBQ^*INDEX*^, occupational burnout syndrome composite index.*

**TABLE 2:** The distribution of the variable level of psychological comfort.

Level of psychological comfort	*n*	%
Low	217	39.74%
Medium	154	28.21%
High	174	31.87%
Unknown	1	0.18%

*N = 546.*

**TABLE 3:** Latent variable: Level of psychological comfort.

Level of psychological comfort	Effect (β)				
	
	a	b	Direct (c)	Indirect (ab)	Total (c + ab)
Low	−0.383 (−0.492; −0.273)***	−0.016 (−0.158; 0.126)	0.175 (0.036; 0.314)*	0.006 (−0.048; 0.061)	0.181 (0.053; 0.309)**
Moderate	−0.192 (−0.343; −0.041)*	0.123 (−0.036; 0.282)	0.046 (−0.114; 0.206)	−0.024 (−0.059; 0.012)	0.022 (−0.136; 0.181)
High	−0.401 (−0.522; −0.281)***	0.234 (0.078; 0.39)**	0.129 (−0.029; 0.287)	−0.094 (−0.164; −0.024)**	0.035 (−0.114; 0.185)

*Standardized results of regression analyses performed on the total effect, direct effect, and indirect effect in group of Polish ECD’s (*N* = 546).*

a – effect stress on self-efficacy; b – effect self-efficacy on burnout; c – direct effect stress on burnout; ab – indirect effect stress on burnout via self-efficacy; combined effect stress and self-efficacy on burnout, total (c + ab) – total stress relationship with burnout, including indirect relationships through self-efficacy. ***p < 0.001; **p < 0.01; and *p < 0.05.

The results showed the following relationships. The level of psychological comfort moderated the negative relationship between stress and self-efficacy. Both when the comfort level was high (β_*H*_ = −0.401, *p* < 0.001, LLCI = −0.522; ULCI = −0.281), average (β_*M*_ = −0.192, *p* < 0.05, LLCI = −0.343; ULCI = −0.041), and low (β_*L*_ = −0.383, *p* < 0.001, LLCI = −0.492; ULCI = −0.273). The low level of psychological comfort moderated the positive relationship between stress and burnout (β_*CL*_ = 0.174, *p* < 0.05, LLCI = 0.036; ULCI = 0.314). When the comfort level was low, the strength of the relationship combined compound (including self-efficacy) was greater than in the case of the direct relationship (β_*C+ABL*_ = 0.181, *p* < 0.01; LLCI = 0,053, ULCI = 0,309). The high level of psychological comfort moderated the positive self-efficacy relationship with burnout (β_*H*_ = 0,234, *p* < 0,01, LLCI = 0,078, ULCI = 0.39). The high level of psychological comfort moderated the negative indirect impact of stress on burnout via self-efficacy (β_*H*_ = −0.094, *p* < 0.05; LLCI = −0.164, ULCI = −0.024). The low level of psychological comfort moderated the combined positive effect of stress and self-efficacy on burnout (β_*L*_ = 0.181, *P* < 0.01, LLCI = 0,053, ULCI = 0.309). Taking into account the level of self-efficacy in the relationship between stress and burnout with a low level of psychological comfort strengthened the strength of the relationship between stress and burnout.

### Model 2. The Level of Power-to-Affect as Moderator of Predictors of Occupational Burnout

**FIGURE 2:**
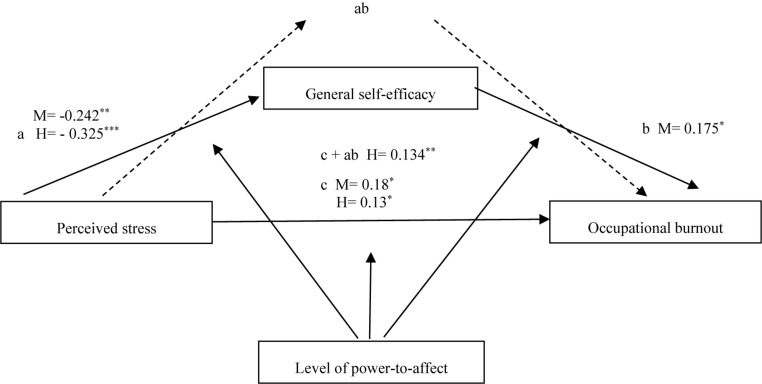
Model 2. Relationships between perceived stress, general self-efficacy and occupational burnout moderated by level of power-to-affect in a group of Polish ECD’s. L = low level of power-to-affect. M = medium level of power-to-affect. H = high level of power-to-affect. ^∗^β is significant at the 0.05 level. ^∗∗^β is significant at the 0.05 level. ^∗∗∗^β is significant at the 0.05 level. For the readability of the graph, the graphic markings of irrelevant paths were omitted.

The sten scores adopted in the interpretation of the GSES results were used to distinguish three groups of respondents due to the latent variable – level of power-to-affect. The group with low level of power-to-affect was composed of results in the range of 1–4 sten score, moderate 5–6 sten score and high 7–10 sten score. Table 4 exhibits the distribution of the variable “level of psychological comfort.” The path analyzing model was proposed to explain the moderating influence of psychological comfort on the relationship between explanatory variables and burnout (see Table 5 and Figure 2).

**TABLE 4:** The distribution of the variable level of power-to-affect.

Power-to-affect	*n*	%
Low	28	5.13%
Moderate	140	25.64%
High	375	68.68%
Unknown	3	0.55%

*N = 546.*

**TABLE 5:** Latent variable: Level of power-to-affect.

Level of power-to-affect	Effect (β)				
	
	a	b	Direct (c)	Indirect (ab)	Total (c + ab)
Low	−0.31 (−0.643; 0.022)	0.303 (−0.061; 0.667)	0.118 (−0.258; 0.494)	−0.094 (−0.249; 0.061)	0.024 (−0.353; 0.401)
Medium	−0.242 (−0.396; −0.089)**	0.175 (0.011; 0.339)*	0.18 (0.017; 0.342)*	−0.042 (−0.091; 0.006)	0.137 (−0.025; 0.299)
High	−0.325 (−0.413; −0.237)***	−0.021 (−0.127; 0.086)	0.13 (0.025; 0.235)*	0.007 (−0.028; 0.041)	0.137 (0.038; 0.236)**

*Standardized results of regression analyses performed on the total effect, direct effect, and indirect effect in group of Polish ECD’s (*N* = 546).*

a – effect stress on self-efficacy; b – effect self-efficacy on burnout; c – direct effect stress on burnout; ab – indirect effect stress on burnout via self-efficacy; combined effect stress and self-efficacy on burnout, total (c + ab) – total stress relationship with burnout, including indirect relationships through self-efficacy. ***p < 0.001; **p < 0.01; and *p < 0.05.

Medium level power-to-affect (β_*M*_ = −0.242, *p* < 0.01, LLCI = −0.396, UCLI = −0.089) and a high level of power-to-affect (β_*H*_ = −0.325, *p* < 0.001, LLCI = −0.413, UCLI = −0.237) moderated the negative relationship of stress with self-efficacy. In the case of a low power-to-affect level, there was no relationship. The average level of power-to-affect moderated the positive self-efficacy relationship with burnout (β_*M*_ = −0.175, *p* < 0.05, LLCI = 0.011, UCLI = 0.339). High level power-to-affect (β_*H*_ = 0.13, *p* < 0.05, LLCI = 0.025; ULCI = 0.235) and a medium level of power-to-affect (β_*M*_ = 0.18, *p* < 0.05, LLCI = 0.017; ULCI = 0.342) moderated the positive effect of stress on burnout. The indirect effect of stress on burnout (through self-efficacy) was not significant at each power-to-affect level. The high level of power-to-affect moderated the total positive impact of stress on burnout (β_*H*_ = 0.137, LLCI = 0.038, ULCI = 0.236).

## Discussion

Two models were tested taking into account the moderating effect of latent variables on the relationships: perceived stress – self-efficacy – level of occupational burnout in the emergency dispatchers and call-takers occupational group. First, the changes in relationships caused by the introduction to the model of the level of psychological comfort, operationalized as three levels of perceived stress intensity, were checked. Then, the changes caused by the introduction of the power-to-affect level, operationalized as three levels of intensity of the personal factor of self-efficacy, were examined.

In line with the hypotheses, the study confirmed the existence of negative relationships between perceived stress (assessment of the current situation) and self-efficacy (a personal trait). Both perceived stress and self-efficacy hide specific cognitive beliefs. A low level of comfort means the psychological tension accompanying the beliefs that a person is helpless in coping with unpredictable, uncontrollable and overwhelming life events (Cohen et al., 1983, p. 387). Self-efficacy is a generalized belief about the ability to deal with adversity. The negative relationship between the variables is consistent with the COR theory: the threat of losing of sense of self-efficacy triggers stress processes to inhibit negative reactions. If these processes fail and the loss of self-efficacy is not stopped, the sense of perceived stress increases. According to the Job Demands – Resources Model: personal resources can buffer energy processes and, as a result, reduce the level of perceived stress. Workers with a high personal factor of self-efficacy may perceive external stressors as being less threatening. They may be able to manage stressful demands and could prevent the emergence of burnout (Schwarzer and Hallum, 2008). Ivancevich and Matteson (2002) reported that persons with high levels of self-efficacy feel confident in their abilities and are more likely to perceive potential stressors as challenges and opportunities, rather than threats and losses. In contrast, individuals with low levels of self-efficacy are less confident in their abilities and in the successful completion of tasks.

Regarding to self-efficacy – stress relationships, including the moderating influence of three levels of psychological comfort and three levels of power-to-affect in this study confirmed the assumed dependence and showed its relationship with the level of severity of moderators. Regardless of the level of stress, i.e., the number of negative beliefs about controlling and counteracting current stressful life events, the self-efficacy as personal factor acts in opposition to perceiving stress. However, taking into account the differences in the intensity of the abovementioned personality trait, weak beliefs about self-efficacy no longer constituted a significant counterbalance to the currently perceived life stress.

The analysis of correlation revealed that the positive relationship between stress and occupational burnout, known from the literature, turned out to concern only one dimension of burnout in the studied group: loss of professional effectiveness. This result differs from that obtained in the teachers’ research, in which the level of perceived stress co-occurred with increasing emotional exhaustion and increasing negative changes in social relations (Teles et al., 2020). According to the JD-R model, a positive relationship between stress and burnout should be initiated by an increase in exhaustion and energy depletion (Maslach et al., 2001), because professional work involves people on the energy level, leading to the depletion of their psychophysical resources. Especially when the work is performed with great engagement, which is an accompanied by high level of stress. According to COR theory, the loss of any resources, including the sense of professional effectiveness, triggers adaptation processes to stop it. This explains the negative relationship between the above-mentioned variables: stress and loss of professional effectiveness. Taking into account the moderating effect of psychological comfort revealed a relationship between stress and burnout that had not previously appeared in the correlation analysis. The analysis revealed that the positive relationship between stress and burnout occurred only among participants with a low level of psychological comfort (high level of stress), so it was not a proportional relationship. Taking into account the moderating effect of power-to-affect also showed a positive relationship between stress and burnout. It occurred in people who had a high and medium level of power-to-affect, so contrary to what we assumed in the hypotheses. Additionally, in people with a high level of power-to-affect, a positive relationship between stress and burnout occurred after taking into account the combined effect of perceived stress and self-efficacy to burnout. It can therefore be concluded that the introduction of latent variables to the models revealed relationships not visible in the correlation analysis and confirmed their compliance with data from the literature. It can also be seen that the relationships between the discussed variables are complex and not proportional. In addition, it turned out that in the case of people with a high self-efficacy level, taking into account the above-mentioned variable in the stress-burnout relationship did not result in the expected changes. Thus, the hypothesis that a high level of power-to-affect should weaken the relationship between stress and burnout has not been confirmed. If the power-to-affect level is treated as an indicator of resourcefulness in coping with tasks, challenges and stress at work (Shoji et al., 2015), then, according to the JD-R model, people with high power-to-affect should work with greater commitment, and the resource should protect them from burnout. To explain the inconsistency of results using COR theory, it should be stated that people with a higher level of comfort in the face of very burdensome working conditions that currently take place in Polish ECDs centers experience a greater risk of losing their resources, which increases their stress level and thus leads to burnout. Especially if the work takes place in extreme conditions caused by the COVID-19 pandemic.

The negative relationship between self-efficacy and burnout was not confirmed, the correlation analysis revealed a negative relationship only in the case of the loss of professional effectiveness and, contrary to assumptions, a positive relationship with the dimension of job disappointment. Even more so, the hypothesis that the strength of the negative relationship: self-efficacy – burnout is inversely proportional to the level of psychological comfort and directly proportional to power-to-affect was not confirmed. This result is contrary to the one derived from the study of Polish firefighters, in which the personal factor of self-efficacy changed the direction and strength of relationship between perceived stress and PE, sense of personal inefficacy and disillusion (3 out of 4 dimensions of burnout; Makara-Studzińska et al., 2019). In people with a high level of psychological comfort (low level of stress), we observed a positive self-efficacy relationship with burnout. ECD’s with a high intensity of the aforementioned personality trait revealed a high degree of occupational burnout, although according to the literature it should be the other way around. This means that people who at the time of the study were relaxed, calm and attentive, coping well with problems and at the same time having the ability to organize the activities necessary to achieve goals turned out to be more burned out. Taking into account the differences in the level of self-efficacy, people with average intensity of the mentioned trait also revealed a higher degree of burnout compared to the others, so an opposite relationship to the expected one appeared. The average level of power-to-affect revealed a positive self-efficacy relationship with burnout. The interrelationships between the discussed variables assumed in the hypotheses were confirmed in the case of an indirect relationship between stress and burnout via self-efficacy among participants with a high level of psychological comfort. Here, taking into account the self-efficacy personality trait reversed the direction of the stress-burnout relationship. Meanwhile, in studies of Polish firefighters, regardless of differences in the level of perceived stress, people with a lower level of self-efficacy showed more intense signs of emotional exhaustion (Makara-Studzińska et al., 2019).

An important issue is the very high level of occupational burnout identified in the ECD’s group and its possible causes. It is known from the literature that ECD is a profession with a high risk of stress and burnout (Baseman et al., 2018; Smith et al., 2019). However, the results obtained in our study were higher than those obtained in 2018 in the American ECD group, where the burnout criterion was met by 32% of respondents (Boland et al., 2018). Similar data from other countries were even lower, although significantly higher than in the general population (Boland et al., 2018). There is an immediate supposition that there may be two interrelated causes behind the aforementioned differences. The first is the fact that the study was conducted during the COVID-19 pandemic, the second is that the ECD system in Poland is under development. Both of these reasons should be treated together. The system, which was insufficiently organized in formal and legal terms, was additionally ineffective in the face of the pandemic’s challenges, leading to the burnout of a large number of workers in a short time. This is also reflected in the high rotation, ECD in Poland works in the profession on average 3–4 years (Klasa, 2020). A study conducted in Italy among a group of nurses working during the COVID-19 pandemic showed an equally high level of burnout. The symptoms of the syndrome were diagnosed in 68% of respondents (Damico et al., 2020). Similar trends are noticeable in the results concerning employees of emergence services from different countries of the world. The results of a study conducted in France showed a sharp increase in the number of incoming emergency calls per hour, the number of ECD’s involved in a call, and the duration of a call during a pandemic (Penverne et al., 2020). The authors suggest taking specific organizational solutions helpful in times of mass disasters. The first analyzes carried out in the countries affected by the pandemic show the need to improve the methods of managing emergency services by the employees of ECD’s centers in order to better manage human resources (Rashed et al., 2021).

### Indications for Preventive Actions

The European Framework on Safety and Health at Work of European Union obliges employers to protect employees against workplace hazards (Chirico, 2017b). The occupational burnout syndrome has not yet been officially accepted as an occupational disease in EU countries. However, in 9 EU countries, burnout may be acknowledged as an occupational disease, which allows workers to be compensated the health loss (Laštovková et al., 2018). There are still difficulties in identifying psychosocial factors for risk of burnout. There is no clear individual diagnosis of burnout and there are problems with differentiating burnout from other mental disorders: depression, anxiety and stress. Despite the lack of sufficiently accurate methods of measuring burnout and the difficulties in defining it, steps are taken in many countries to prevent this phenomenon. Most of created programs include interventions aimed at the employee. Secondly the program also includes interventions focused on organizational changes and also combined program that include both of the mentioned interventions (Awa et al., 2010). Personal interventions are aimed at increasing the employee’s professional competences, coping skills, using social support and performing relaxation exercises. Interventions aimed at organizing workplace serve to reduce the demand for work, increase the sense of work control and employee participation in decision making, remove the effort reward imbalance. Organizational changes also contribute to the growth of sense of belongings to the community, strong relationships, and work-life balance. Participation in the programs increases the employees’ sense of self-efficiency at work. Along with the proposed new definition of burnout, WHO plans to develop evidence-based guidelines on mental well-being in the workplace (World Health Organization [WHO], 2019). It should be emphasized that the negative health effects of burnout are not limited to mental health alone. Inflammatory processes caused by low cortisol levels in burned out workers may contribute to impaired immune functions, pathogenesis of chronic diseases and sleep disorders (Melamed et al., 2006). Physiological changes may secondarily increase employees’ feelings of emotional exhaustion and weariness. These facts indicate the importance of undertaking preventive interventions of an interdisciplinary nature, both psychological, organizational and medical. Due to the objectively high risk of burnout in the ECD’s professional group, programs are created around the world to counter the phenomenon by strengthening selected personal resources (e.g., Linos et al., 2019). The results obtained by us may contain suggestions for preventive measures. It can be seen that strengthening the self-efficacy personality trait may be a way to protect against burnout, however, when the level of the current perceived life stress is not high. In such a case, it seems that the first step should be changes aimed at discovering the predictors of stress in order to exclude them. The self-efficacy personality trait does not always act as a buffer protecting the employee against burnout. It may intensify some of its dimensions, which may be fostered by the level of stress currently experienced. It seems right to treat resources as interrelated factors in order to more accurately predict the expected changes in their impact on stress and burnout.

### The Limitations

In order to explain the cause-and-effect relationships concerning the discussed issue, it seems helpful to conduct longitudinal studies. The theoretical background is COR theory, which allows to show dynamic changes in relations – resources – stress – burnout. It is also reasonable not to limit research tools only to self-report methods but to use objective indicators to measure stress and burnout.

## Conclusion

The obtained results partially confirmed the research hypotheses and answered research questions. Taking into account the differences in the intensity of explanatory variables showed their moderating effect, which often turned out to be different from the assumed one and obtained in the research of other authors. It revealed relationships that might otherwise have been missed. The study provided a wealth of information about relationships: perceived stress – self-efficacy and burnout. First, it showed a relationship characterized by proportional relationships between the variables and consistent in its direction and strength with data from the literature. We are talking about a negative relationship between perceived stress and self-efficacy, which persists despite taking into account the differences in the intensity of the explanatory variables. Second, although the correlation analyzes did not indicate it, taking into account the moderating effect of latent variables allowed us to see the expected relationships between the variables. It is about changing the direction of the relationship between perceived stress and burnout via self-efficacy, which occurred only in people with a high level of psychological comfort (low level of stress). Only in this case we observed a self-efficacy buffering effect on the relationship between stress and burnout. Third, considering the influence of the moderators revealed accounts that turned out to be opposite to the data from the literature. We are talking about a positive self-efficacy relationship with burnout, which occurred in people with a high level of psychological comfort (low level of stress) and in people with an average level of power-to-affect (average level of self-efficacy).

## Data Availability Statement

The raw data supporting the conclusions of this article will be made available by the authors, without undue reservation.

## Ethics Statement

The study protocol was approved by the Bioethics Commission at Jagiellonian University Medical College (decision No. 1072.6120.23.2017) and was carried out in accordance with the recommendations of the APA Ethics Code. The patients/participants provided their written informed consent to participate in this study.

## Author Contributions

All authors listed have made a substantial, direct and intellectual contribution to the work, and approved it for publication.

## Conflict of Interest

The authors declare that the research was conducted in the absence of any commercial or financial relationships that could be construed as a potential conflict of interest.

## Publisher’s Note

All claims expressed in this article are solely those of the authors and do not necessarily represent those of their affiliated organizations, or those of the publisher, the editors and the reviewers. Any product that may be evaluated in this article, or claim that may be made by its manufacturer, is not guaranteed or endorsed by the publisher.
